# Comprehensive analysis of the prognostic and role in immune cell infiltration of MSR1 expression in lower‐grade gliomas

**DOI:** 10.1002/cam4.4603

**Published:** 2022-02-10

**Authors:** Qiankun Ji, Kai Huang, Yuan Jiang, Kunjian Lei, Zewei Tu, Haitao Luo, Xingen Zhu

**Affiliations:** ^1^ Department of Neurosurgery The Second Affiliated Hospital of Nanchang University Nanchang Jiangxi China; ^2^ Institute of Neuroscience Nanchang University Nanchang Jiangxi China

**Keywords:** lower‐grade gliomas, macrophage scavenger receptor 1, tumor microenvironment, tumor‐infiltrating immune cells

## Abstract

**Background:**

The therapeutic effects of conventional treatment on gliomas are not promising. The tumor microenvironment (TME) has a close association with the invasiveness of multiple types of tumors, including low‐grade gliomas (LGG). This study aims to validate the prognostic and immune‐related role of macrophage scavenger receptor 1 (*MSR1*) in LGG patients.

**Methods:**

Data in this study were obtained from public databases. The differential expression of *MSR1* was analyzed in LGG patients with different clinicopathological characteristics. Kaplan–Meier survival analysis, a time‐dependent receiver operating characteristic (ROC) curve, and Cox regression analysis were used to assess the prognostic value of *MSR1*. Differentially expressed genes (DEGs) were screened between the high and low expression groups of MSR1. Gene Ontology (GO) and Kyoto Encyclopedia of Genes and Genomes (KEGG) were used to annotate the function of these DEGs. Hallmark gene sets were identified based on *MSR1* by Gene Set Enrichment Analysis (GSEA). Difference analysis and correlation analysis were used to study the relationship between *MSR1* and TME‐related scores, tumor‐infiltrating immune cells (TIICs), immune‐related gene sets, and immune checkpoints (ICPs). The single‐cell sequencing data were processed to identify the cell types expressing *MSR1*. The quantification of TIICs in TME was calculated by single‐sample gene set enrichment analysis (ssGSEA). The differential expression of *MSR1* in LGG and control brain tissues was verified by experiments.

**Results:**

There were significant differences in the expression level of *MSR1* in different types of tissues and cells. *MSR1* has a high prognostic value in LGG patients and can be used as an independent prognostic factor. MSR1 is closely related to TME and may play an important role in the immunotherapy of LGG patients.

**Conclusions:**

The result of our study demonstrated that *MSR1* is an independent prognostic biomarker in LGG patients and may play an important role in the TME of LGGs.

## Graphical abstract

The tumor microenvironment (TME) has a close association with the invasiveness of multiple cancers, including low‐grade gliomas (LGGs). The aim of this study is to validate the prognostic and immune‐related role of macrophage scavenger receptor 1 (MSR1) in LGGs.

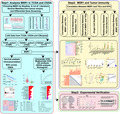

## 1 INTRODUCTION

The characteristics of gliomas include high invasiveness, the propensity to relapse, and lethality.^1^ LGGs comprise diffuse LGGs (WHO II) and metaplastic gliomas (WHO III).^2^ Surgical resection, adjuvant chemotherapy, postoperative radiotherapy, and immunotherapy form the standard treatments for gliomas.^3^, ^4^, ^5^ Despite advances in techniques and equipment for glioma surgery, the overall survival (OS) of glioma patients remains poor.^6^ The main challenges include therapeutic resistance and tumor recurrence.^7^ In recent years, immunotherapy has become a very promising treatment for multiple types of cancers. More and more studies show that tumor‐infiltrating leukocytes are related to therapeutic effects and cancer prognosis.^8^, ^9^, ^10^, ^11^, ^12^ As the prognosis of LGGs and glioblastomas (GBM) differ significantly, this study mainly focused on LGGs.

In addition to tumor cells, tumor tissue also contains other cellular components, such as immune cells and stromal cells. There is an increasing amount of evidence demonstrating that TME is related to a variety of biological behaviors of cancer cells,^13^, ^14^, ^15^, ^16^, ^17^ which has become a research hotspot.^18^ The mechanism underlying TME formation is very complex. As an important part of TME, TIICs account for about 30% of the tumor mass^19^ and are considered to play an important role in the tumorigenesis and development of a variety of tumors. Classically activated M1 macrophages promote an anti‐tumor response, while tumor‐associated macrophages (TAMs) have an immunosuppressive role in most types of tumors.^20^, ^21^, ^22^ TAMs restrain a series of immune responses and form an immunosuppressive microenvironment by preventing T cell proliferation, promoting T cell apoptosis, inhibiting cytotoxic T cell response, and promoting the activation of other immunosuppressive cells.^23^, ^24^ Several key inflammatory mediators, confirmed by many studies, actively participate in the process of tumor progression.^25^, ^26^, ^27^ Macrophages are the main component of the leukocyte population and make major contributions to host immunity.^28^ A high frequency of M2‐polarized TAMs characterized by the expression of *MSR1* is associated with poor prognosis in various cancers.^29^ Several metabolic processes of macrophages are related to *MSR1*, such as adhesion and phagocytosis.^30^ In addition, *MSR1*+ TAMs represent a reliable prognostic indicator in esophageal squamous cell carcinoma and breast cancer.^31^, ^32^ The copy number variation (CNV) of *MSR1* also plays a role in oncogenesis.^33^ The base sequence of *MSR1* is abnormally enriched in DNA repeats, with a 36–38‐bp microsatellite repeat element.^34^

In this study, we evaluated the prognostic value of *MSR1* in pan‐cancer and found that it has outstanding prognostic value in LGG patients, which prompted us to conduct further study. Extensive bioinformatics analyses were performed to explore the relationship between *MSR1* and different clinicopathological characteristics in LGG patients. The results of univariate and multivariate Cox regression analysis demonstrated that *MSR1* could be used as an independent prognostic factor in LGG patients. Subsequently, we constructed a nomogram to further explore the prognostic value of *MSR1*. The results of GO and KEGG enrichment analysis suggest that *MSR1* may play a role in tumor immune‐related functions and pathways. Then, we calculated the TME‐related scores for each sample in the TCGA cohort and analyzed the relationship between them and *MSR1*. Besides, we also studied the relationship between *MSR1* and immune‐related gene sets and TIICs. Furthermore, the associations of *MSR1* with the ICPs (inhibitory molecules that play key roles in maintaining autoimmune tolerance and regulating the duration and amplitude of the physiological immune response) were also explored to elucidate the potential effect of immunotherapy in LGG patients. Lastly, the results of single‐cell sequencing data processing results suggested that *MSR1* was mainly expressed in macrophages and monocytes in LGG tissue.

## 2 MATERIALS AND METHODS

### 2.1 Raw data and preprocessing

Pan‐cancer expression and corresponding clinical data were obtained from the UCSC Xena data portal. Gene expression data (FPKM) were downloaded from The Cancer Genome Atlas (TCGA) database. Related expression data of two validation cohorts (CGGAseq1 and CGGAseq2) and the corresponding clinical profiles were retrieved from the Chinese Glioma Genome Atlas (CGGA) database. Single‐cell sequencing data (GSE84465) of LGG patients were obtained from the Gene Expression Omnibus (GEO) database. Additionally, immune‐associated gene sets and key ICPs were identified based on Auslander's study.^35^ The criteria for LGG patients to be included in this study: (a) patients without survival status or time, or survival time <30 days; (b) patients without WHO grade or gene expression data. The clinical information of the included patients is shown in Table 1.

**TABLE 1:** Basic information of low‐grade glioma (LGG) patients

Features	Total (*n* = 1070)	TCGA cohort (*n* = 480)	CGGAseq1 cohort (*n* = 420)	CGGAseq2 cohort (*n* = 170)
Overall survival (years)				
Median (range)	2.57 (0.10–17.60)	1.62 (0.10–17.60)	3.95 (0.14–13.78)	5.96 (0.18–13.18)
<5	769 (71.9%)	423 (88.7%)	264 (62.9%)	82 (48.2%)
≥5	301 (28.1%)	57 (11.3%)	156 (37.1%)	88 (51.8%)
Age (years)				
Median (range)	40 (10–87)	41 (14–87)	40 (11–72)	39 (10–74)
<41	539 (50.4%)	233 (48.5%)	211 (50.2%)	95 (55.9%)
≥41	530 (49.5%)	247 (51.5%)	208 (49.5%)	75 (44.1%)
NA	1 (0.1%)	0 (0.0%)	1 (0.2%)	0 (0%)
Gender				
Male	602 (56.3%)	218 (45.4%)	185 (44.0%)	105 (61.8%)
Female	468 (43.7%)	262 (54.6%)	235 (56.0%)	65 (38.2%)
WHO grade				
II	501 (46.8%)	232 (48.3%)	172 (41.0%)	97 (57.1%)
III	569 (53.2%)	248 (51.7%)	248 (59.0%)	73 (42.9%)
IDH mutation status				
Mutant	805 (75.2%)	392 (81.7%)	288 (68.6%)	125 (73.5%)
Wild	223 (20.8%)	85 (17.7%)	94 (22.4%)	44 (25.9%)
NA	42 (3.9%)	3 (0.6%)	38 (9.0%)	1 (0.6%)
1p/19q codeletion status				
Non‐codeletion	693 (64.8%)	323 (67.3%)	257 (61.2%)	113 (66.50%)
Codeletion	337 (31.5%)	157 (32.7%)	125 (29.8%)	55 (32.4%)
NA	40 (3.7%)	0 (0%)	38 (9.0%)	2 (1.2%)
MGMT status				
Methylated	681 (63.6%)	397 (82.7%)	200 (47.6%)	84 (49.4%)
Unmethylated	282 (26.4%)	83 (17.3%)	129 (30.7%)	70 (41.2%)
NA	107 (10%)	0 (0%)	91 (21.7%)	16 (9.4%)

### 2.2 Tumor samples

Tissue samples (23 tissue samples: 7 WHO III glioma samples, 11 WHO II glioma samples, and 5 non‐neoplastic brain tissues [controls] from epilepsy surgery patients) obtained from 2017 to 2020 were retrieved from The Second Affiliated Hospital of Nanchang University and stored at −80°C.

### 2.3 Correlation of MSR1 and clinicopathological characteristics in LGG patients

Differential expression of *MSR1* between six different clinicopathological characteristics, including gender, age, WHO grade, isocitrate dehydrogenase (IDH) mutation, 1p19q co‐deletion, and O6‐methylguanine‐DNA methyltransferase (MGMT)**,** among LGG patients, were analyzed by the R package “beeswarm” (version 0.2.3). Survival analysis was conducted by the R packages “survival” (version 3.2–7) and “survminer” (version 0.4.8). In addition, the ROC curve was plotted to predict the 1‐, 3‐, and 5‐year OS of LGG patients in the TCGA, CGGAseq1, and CGGAseq2 cohorts using the R package “survivalROC” (version 1.0.3). Univariate Cox regression analysis of six clinicopathological characteristics and *MSR1* was conducted by the R package “survival”. Multivariate Cox regression analysis was used to discover the independent prognostic factors.

### 2.4 Construction and validation of a nomogram

We constructed a nomogram in the TCGA cohort, including age, WHO grade, IDH mutations, 1p19q co‐deletion, and *MSR1*. Gender and MGMT were not used to construct the nomogram because they were not independent prognostic factors in any of the three cohorts. To verify the accuracy of the nomogram, we constructed calibration plots in the TCGA, CGGAseq1, and CGGAseq2 cohorts using the R packages “rms” (version 6.1–1), “foreign” (version 0.8–80), and “survival”.

### 2.5 Functional enrichment analysis

The R package “limma” (version 3.46.0) was used to identify differentially expressed genes (DEGs; based on |log2 [FC]| > 1 and *p* < 0.05) between the high and low expression groups of *MSR1* in LGG patients. GO and KEGG enrichment analysis based on the DEGs were conducted using the R packages “clusterProfiler” (version 4.2.0), “enrichplot” (version 1.10.2), and “ggplot2” (version 3.3.3). Gene‐set enrichment analysis software (GSEA, version 4.0.1, https://www.gsea‐msigdb.org/gsea/index.jsp)^36^ was used to identify relevant Hallmark gene sets of *MSR1* (based on |normalized enrichment score (NES)| > 1.5, normalized *p* < 0.01, and false discovery rate [FDR]‐adjusted *q* < 0.01).

### 2.6 Identification of DEGs based on immune and stromal scores

We calculated the immune score (proportion of immune cells in tumor tissues), stromal score (proportion of immune cells in tumor tissues), and ESTIMATE scores (sum of immune and stromal scores; the higher the ESTIMATE score, the lower the tumor purity) for each sample in the TCGA cohort using the ESTIMATE algorithm (Estimation of Stromal and Immune cells in malignant Tumor tissues using Expression data).^37^ The patients were divided into two groups based on the median value of the immune or stromal scores, and DEGs were identified between the two groups using the R package “limma” with the threshold: log2(FC) > 1 (high vs. low) and FDR < 0.05.

### 2.7 Single‐sample gene set enrichment analysis

Single‐sample gene set enrichment analysis (ssGSEA) was used to calculate the enrichment score of the 29 immune‐associated gene sets obtained from previous studies.^38^, ^39^ Estimating Relative Subsets of RNA Transcripts (CIBERSORT)^40^ method was used to identify human hematopoietic cell types, which relies on a leukocyte gene signature matrix involving 547 genes. The infiltration of TIICs in the TME was quantified by the R package “GSVA”.^41^ The differences in infiltration levels of six types of TIICs between different CNV types of *MSR1* were assessed on the Tumor Immune Estimation Resource website (TIMER, https://cistrome.shinyapps.io/timer/).

### 2.8 Processing of single‐cell RNA sequencing data

The single‐cell RNA sequencing data of LGG patients were processed by the R package “Seurat” (version 4.0.1), using the “NormalizeData” function to normalize the data. A total of 4000 highly variable genes were identified. First, these genes were processed by principal component analysis (PCA) using the “RunPCA” function. Next, a k‐nearest neighbor graph was constructed using the “FindNeighbors” function. Cells were optimally clustered using the “FindClusters” function. Thereafter, the “sTNE” function was used to identify the cell types. Finally, the “FeaturePlot” function was used to display the gene expression.

### 2.9 Acquisition and analysis of ICPs

We obtained 19 ICPs (*ADORA2A, CD274, CD276*, and so on) from the previous study.^35^ The expression data of ICPs were extracted from the TCGA cohort, and the differential expression of ICPs between high and low expression groups of *MSR1*were performed on the Sanger box website (http://sangerbox.com/Tool). In addition, we used the R packages “ggplot2”, “ggpubr” (version 0.4.0), and “ggExtra” (version 0.9) to analyze Spearman's correlation between *MSR1* and ICPs.

### 2.10 Quantitative real‐time PCR

We used TRIzol reagent (BSC52M1, Life Technologies) to extract total RNA from LGG tissue and control brain tissue. Next, 1 μg total RNA was reverse transcribed into cDNA by using a PrimeScript reverse transcription kit (PrimeScript™ RT‐PCR kit, RR014A, Takara), which included RT primer mix (oligo dT primer and random 6 mers). FastStart Universal SYBR Green Master (04913850001, Roche Diagnostics, Basel, Switzerland) was used to conduct qRT‐PCR. The information of primers for *MSR1* and *GAPDH* are listed in Table S1. Predenaturation at 95°C for 10 min, denaturation at 95°C for 5 s, annealing at 60°C for 30 s, extension at 72°C for 30 s, a total of 40 cycles were carried out, and finally, the dissolution curve was prepared at 65–95°C. *GAPDH* was selected as the internal reference gene, three replicates were made for each sample, and the differential expression of *MSR1* was analyzed quantitatively by the 2^−ΔΔCT^ method. The CT values of *MSR1* and *GAPDH* ranged from 16 to 26. The final analysis results are presented in the form of a histogram, which was plotted by GraphPad Prism 8 software (version 8.0.2)

### 2.11 Western blot analysis

LGG tissue and control brain tissue were lysed by high‐efficiency radioimmunoprecipitation assay (RIPA) buffer with protease inhibitor (R0020, Solarbio). Protein (20 μg protein to each lane) were separated and transferred onto PVDF membrane (ISEQ00010, Millipore) by 10% SDS‐PAGE, which was prepared from 30%PAGE pre‐solution (A1010, Solarbio), 1.5 M Tris–HCL buffer (PH = 8.8) (T1010, Solarbio), 1 M Tris–HCL buffer (PH = 6.8) (T1020, Solarbio), 10%SDS solution (S1010, Solarbio), 10%APS solution (A1030, Solarbio), TEMED (T8090, Solarbio), and double‐distilled water in different proportions. The PVDF membrane was sealed with a mixture (concentration 3%) of bovine serum albumin (A8020, Solarbio) and TBST. TBST is prepared from Tween‐20 (T8220, Solarbio), sodium chloride (CAS:7647‐14‐5, Sigma‐Aldrich), TRIS (T8060, Solarbio), and double‐distilled water in different proportions. After sealing, the PVDF membrane was washed with TBST three times, 15 min each time, and then incubated with *MSR1* (Abcam, ab123946, 1:2000 dilution) and *GAPDH* (Abcam, ab8245, 1:2000 dilution) primary antibodies at 4°C for one night, and then washed with TBST three times again. After that, the PVDF membrane was incubated with a second antibody (Abcam, goat anti‐rabbit antibody, ab6721 1:5000 dilution) for 2 h, and then washed with TBST three times. Finally, the protein bands were detected using enhanced ECL substrate (P180196, Thermo Fisher Scientific) and a GV6000M imaging system (GelView 6000pro). Western blot analysis was repeated three times in this study.

### 2.12 Statistical analysis

Wilcoxon rank‐sum test was used to explore the comparisons between two groups. Comparisons among three or more groups were performed using Kruskal–Wallis tests. The correlations between continuous variables were performed using Spearman's correlation analysis. Kaplan–Meier method was used to explore survival analysis and log‐rank tests were used to identify significant differences. Univariate and multivariate Cox regression was used to analyze *MSR1* expression and identify it as an independent prognostic factor in the TCGA and CGGA cohorts. The specificity and sensitivity of using *MSR1* expression to predict survival were determined using a ROC curve analysis. All statistical tests were two‐sided and *p* < 0.05 was statistically significant. In this study, the version of R software is 4.0.3 (https://www.r‐project.org/).

## 3 RESULTS

### 3.1 The workflow of the study

**FIGURE 1:**
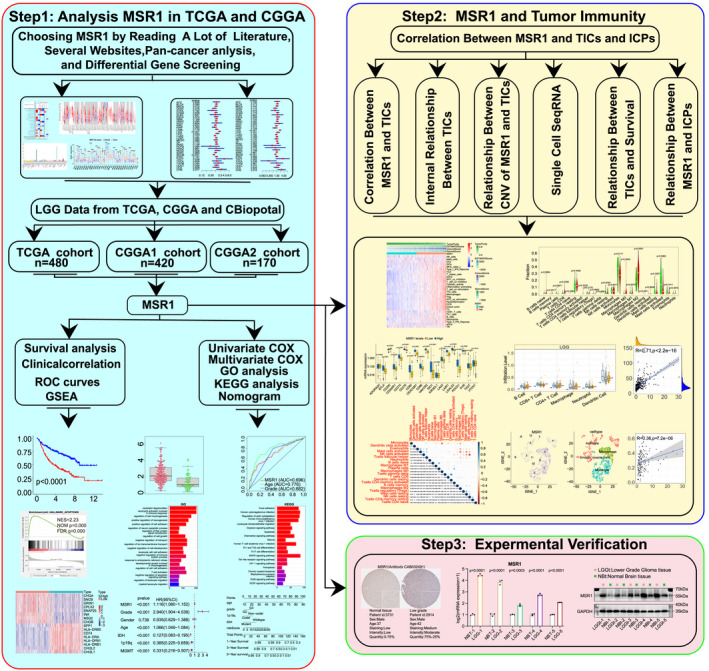
Design of the study. Step 1: Data were downloaded from various websites and databases. Pan‐cancer survival analysis of *MSR1* was conducted. In addition, further study was conducted in the TCGA, CGGAseq1, and CGGAseq2 cohorts (clinical correlation, survival analysis, GO, and KEGG enrichment analysis, and so on). Step 2: Immunological analysis of *MSR1* (including TME, immune‐associated gene sets, TIICs, and ICPs). Step 3: Experimental validation of the differential expression of *MSR1* between LGG tissue and normal brain tissue. GO, Gene Ontology; ICP, immune checkpoint; KEGG, Kyoto Encyclopedia of Genes and Genomes; LGG, low‐grade glioma; TIICs, tumor‐infiltrating immune cells; TME, tumor microenvironment

Step 1: We downloaded raw data from various websites and databases, and then process and analyze the raw data step by step. Step 2: Based on step 1, we explored the association between MSR1 and TME, immune‐associated gene sets, TIICs and ICPs. Step 3: After steps 1 and 2, we verified the differential expression of MSR1 between LGG tissue and normal brain tissue through experiments (Figure 1).

### 3.2 Extensive analysis of *MSR1* in pan‐cancer

**FIGURE 2:**
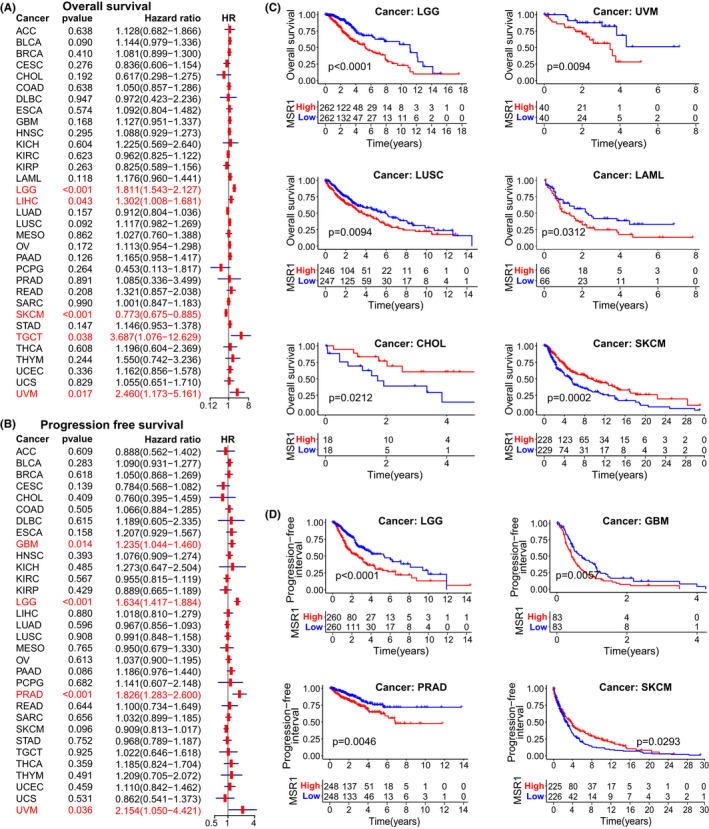
Prognostic value analysis of *MSR1* in pan‐cancer. (A) Forest plot reveals the HR of *MSR1* predicting the OS for different cancer species in the univariate Cox regression analysis. Cancer species with significant statistical differences were marked red and LGG was included (*p* < 0.001). (B) In the univariate Cox regression analysis, the HR of *MSR1* in predicting the PFS for different cancer species is displayed in the forest plot. Cancer species with significant statistical differences were also marked red, including LGG (*p* < 0.001). The results of univariate Cox regression analysis indicated that *MSR1* may be a risk factor for LGG patients. Risk factor: HR > 1; protective factor: HR < 1. (C, D) *MSR1* predicts OS and PFS for cancer patients by the Kaplan–Meier method. Cancer species with significant statistical differences (*p* < 0.05) were displayed in the diagram. The OS and PFS of LGG patients were significantly different between high and low expression groups of *MSR1* (*p* < 0.0001). HR, hazard ratio; LGG, low‐grade glioma; OS, overall survival; PFS, progression‐free interval

By analyzing the differential expression of *MSR1* in different types of tumor tissues and corresponding normal tissues on the GEPIA (http://gepia.cancer‐pku.cn/detail.php), Oncomine (https://www.oncomine.org/resource/login.html), and TIMER websites, we found that the expression level of *MSR1* was higher in most types of tumor tissues than that in corresponding normal tissues (Figure S1A–C). Similar results were found in the TCGA cohorts (Figure S1D). Subsequently, we analyzed the differential expression of *MSR1* in different types of normal tissues and cells on the Human Protein Atlas website (HPA, https://www.proteinatlas.org/). At the level of gene transcription and translation, there are some differences in the expression of *MSR1* in different types of normal tissues and cells (Figure S2A–C). After that, a pan‐cancer survival analysis (involving overall survival [OS], progression‐free survival [PFS], disease‐specific survival [DSS], and disease‐free interval [DFI]) was performed to explore the prognostic value of *MSR1* using Cox regression analysis, Kaplan–Meier method, and the log‐rank test. The results suggested that *MSR1* could be used as a prognostic biomarker in multiple types of tumors (Figure 2A–D, Figure S3A–D), including LGG.

### 3.3 Associations between *MSR1* and clinicopathological characteristics

**FIGURE 3:**
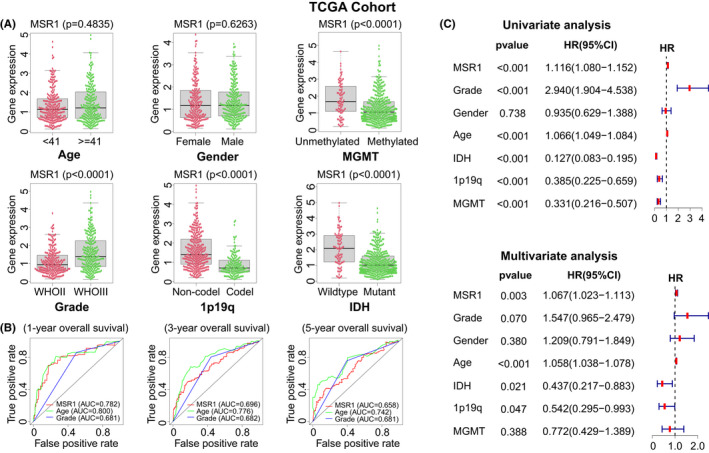
Clinical correlation analysis of *MSR1* in LGG patients in the TCGA cohort. (A) Differential expression of *MSR1* among six clinicopathological characteristics (gender, age, WHO grade, IDH mutation status, 1p19q co‐deletion status, and MGMT). In addition to age and gender, there were significant statistical differences in the other four clinicopathological characteristics with *p* < 0.0001. The red and green dots represent *MSR1* expression levels in different samples. The middle line in the box plot represents the median value of *MSR1* expression, and the upper and lower lines in the box plot represent the upper quartile and lower quartile respectively. There is also a line above the box plot. If the *MSR1* expression level exceeds this line, it is considered an abnormal value. (B) Accuracy of time‐dependent ROC curves of *MSR1*, WHO grade, and age in predicting 1‐, 3‐, and 5‐year OS for LGG patients. The greater the AUC value, the higher the prediction accuracy of clinical characteristics. (C) Univariate and multivariate Cox regression analysis for six clinicopathological characteristics and *MSR1*. The HR and 95%CI of six clinicopathological characteristics and *MSR1* are shown in the forest plot. AUC, area under the curves; LGG, low‐grade glioma; OS, overall survival; ROC, receiver operating characteristic

In the TCGA and CGGA cohorts, we analyzed the differential expression of *MSR1* between different clinicopathological characteristics in LGG patients (Figure 3A, Figure S4A,B). The results showed that the differential expression of *MSR1* among the other five clinicopathological characteristics was statistically more significant than that in gender. To verify the prognostic value of *MSR1*, we conducted an OS analysis of *MSR1* in the CGGAseq1 and CGGAseq2 cohorts and found that the higher the expression of *MSR1*, the lower the OS (Figure S4E,F). The ROC curves of *MSR1*, WHO grade, and age for predicting the 1‐, 3‐, and 5‐years OS of LGG patients suggested that the prognostic value of *MSR1* was considerable. The area under the curves (AUC) of *MSR1* were 0.782, 0.696, and 0.658 in the TCGA cohort (Figure 3B); 0.618, 0.681, and 0.687 in the CGGAseq1 cohort (Figure S4C; 0.707, 0.771, and 0.760 in the CGGAseq2 cohort (Figure S4D). We also performed univariate (TCGA cohort: Table S2; CGGAseq1 cohort: Table S4; CGGAseq2 cohort: Table S6) and multivariate (TCGA cohort: Table S3; CGGAseq1 cohort: Table S5; CGGAseq2 cohort: Table S7) Cox regression analysis for six clinicopathological characteristics and *MSR1* in the three cohorts. The results revealed that *MSR1* could be used as an independent prognostic indicator (HR: 1.067, 95% CI: 1.023–1.113, *p* = 0.003, in TCGA cohort; HR: 1.244, 95% CI: 1.083–1.430, p = 0.002, in CGGAseq1 cohort; HR: 1.214, 95% CI: 1.025–1.437, *p* = 0.025, in CGGAseq2 cohort) (Figure 3C, Figure S4G,H). In addition, we extracted the expression data and corresponding clinical data of LGG patients with *IDH* mutation from TCGA, CGGAseq1, and CGGAseq2 cohorts. Kaplan–Meier survival curves demonstrated that the prognosis of LGG patients with IDH mutation is significantly different between high and low expression groups of *MSR1* (log‐rank test; TCGA cohort: *p* = 0.0482, Figure S5A; CGGAseq1 cohort: *p* < 0.0001, Figure S5D; CGGAseq2 cohort: *p* = 0.0003, Figure S5G). The results of univariate and multivariate COX regression analysis demonstrated *MSR1* could be used as an independent prognostic factor for LGG patients with *IDH* mutation in the three cohorts (Figure S5B,C,E,F,H,I).

### 3.4 Functional enrichment analysis based on DEGs

**FIGURE 4:**
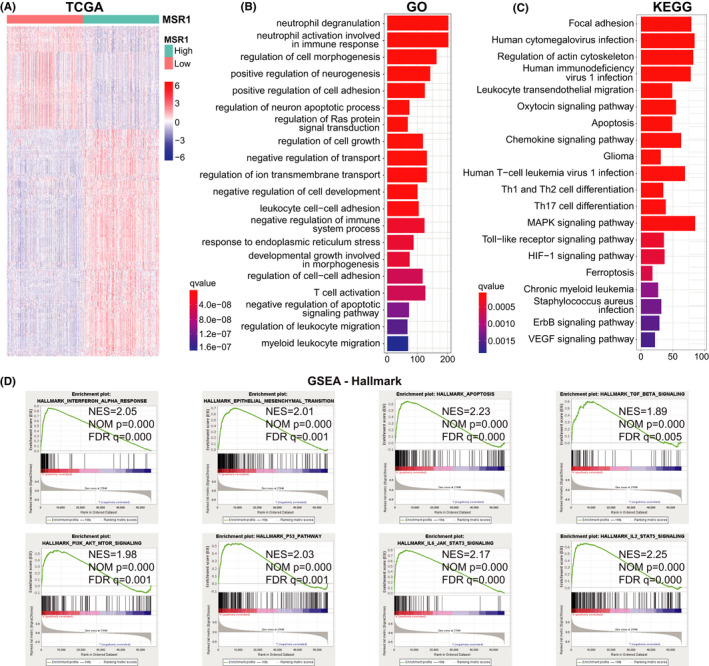
Functional enrichment analysis based on DEGs in the TCGA cohort. (A) DEGs were identified between high and low expression groups of *MSR1* by the Wilcoxon rank‐sum test (FDR < 0.05 and |log2FC| > 2). 311 DEGs were obtained (214 upregulated and 97 downregulated). (B, C) GO and KEGG enrichment analysis based on the DEGs. Some immune‐related pathways and tumor‐related pathways are shown in the histograms. X‐axis: the count of DEGs enriched in the pathway; Y‐axis: the name of the pathway. (D) Eight hallmark gene sets related to *MSR1* were identified by GSEA (nominal *p* < 0.05 and FDR‐adjusted *q* < 0.01). DEGs, differentially expressed genes; GO, gene ontology; GSEA, gene set enrichment analysis; KEGG, Kyoto Encyclopedia of Genes and Genomes; NES, normalized enrichment score

We identified DEGs by comparing the high and low expression groups of *MSR1* in the TCGA (FDR < 0.05 and |log2FC| > 2) and CGGA (FDR < 0.05 and |log2FC| > 1) cohorts. Finally, we obtained 311 DEGs (214 upregulated and 97 downregulated) in the TCGA cohort (Table S8), 594 DEGs (524 upregulated and 70 downregulated) in the CGGAseq1 cohort (Table S9), and 653 DEGs (444 upregulated and 209 downregulated) in the CGGAseq2 cohort (Table S10). Some of them with the most significant difference is displayed in the heatmap (Figure 4A, Figure S6A,B). GO enrichment analysis revealed that the DEGs were closely related to immune‐related function, cell activation, and intercellular adhesion terms (Figure 4B, Figure S6C,D). KEGG enrichment analysis identified the pathways related to DEGs were focused on immune cell differentiation and tumor‐related signaling pathways (Figure 4C, Figure S6E,F). GSEA identified several enriched Hallmark gene sets related to *MSR1*, such as the *P53* pathway, *P13K/AKT/mTOR* signaling pathway, apoptosis, and *TGF‐β* signaling pathway (Figure 4D).

### 3.5 Establishment and validation of a nomogram

**FIGURE 5:**
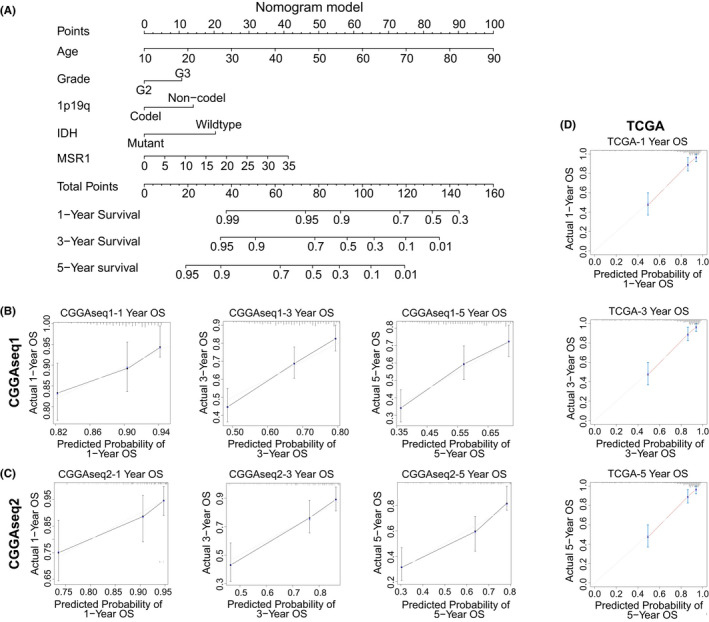
Construction and validation of the nomogram in the TCGA, CGGAseq1, and CGGAseq2 cohorts. (A) The nomogram of clinicopathological characteristics in the TCGA cohort, including *MSR1*, age, WHO grade, IDH mutation status, and 1p19q co‐deletion status. (B–D) Calibration plots were constructed and indicated that the nomogram effectively predicted the 1‐, 3‐, and 5‐year OS for LGG patients in the three cohorts

To further explore the clinical prognostic value of *MSR1*, we constructed a nomogram involving five clinicopathological characteristics and *MSR1* in the TCGA cohort (Figure 5A). In the nomogram, the longer the line, the greater influence of the corresponding factor. The line of *MSR1* indicated that it had a stable effect on the survival prediction. To verify the accuracy of the nomogram, calibration plots were constructed in the three cohorts and suggested that the accuracy of prognosis regarding 1‐, 3‐, and 5‐year OS was very high (Figure 5B–D). The C‐index was calculated to evaluate the discrimination ability of the nomogram. The results showed that the nomogram had good performance (0.849 for the TCGA training cohort, 0.684 for the CGGAseq1 cohort, and 0.716 for the CGGAseq2 cohort).

### 3.6 Role of *MSR1* in TME

We calculated the immune score, stromal score, ESTIMATE score, and tumor purity score for each sample in the TCGA cohort. The higher the immune score, stromal score, and ESTIMATE score, the shorter OS for LGG patients, which is opposite to tumor purity score (Figure S7A, *p* = 0.0067, 0.0004, 0.0086, 0.0086, respectively). In addition, tumor purity score was positively correlated with *MSR1*, while the other three types of scores were negatively correlated with *MSR1* (Figure S7B, *p* < 0.0001, respectively). We obtained 1163 upregulated and 530 downregulated DEGs by taking the intersection between the two groups of DEGs (Figure S7C,D). *MSR1* was one of the upregulated DEGs. The top 8 DEGs are shown in the heatmap (Figure S7E,F).

**FIGURE 6:**
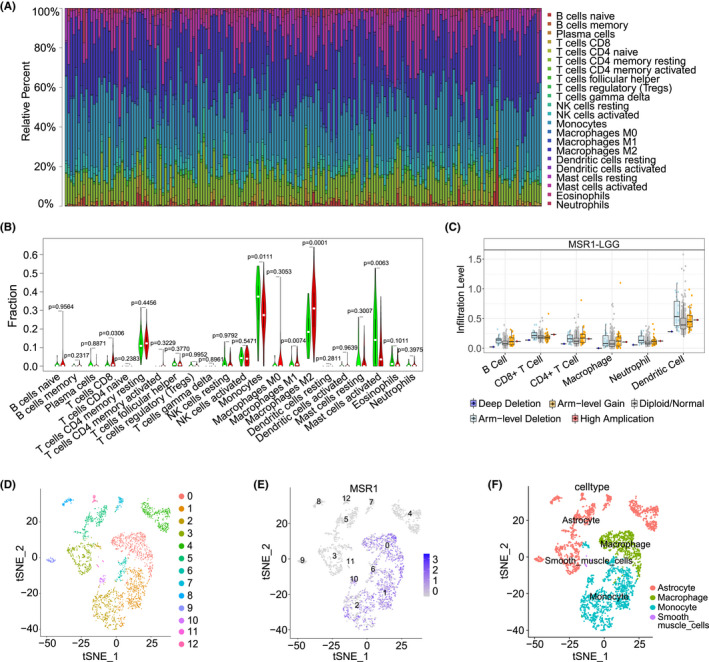
Association between *MSR1* and TIICs in the TCGA cohort. (A) The proportion of different types of TIICs in each LGG patient. Different colors of bar plots represent different types of TIICs, and the height of the bar plot corresponds to the percentage of TIICs. (B) The difference in the proportion of TIICs between high and low expression groups of *MSR1*. The proportion of CT8^+^ T cells, monocytes, macrophages M1, macrophages M2, and activated mast cells was significantly different between the two expression groups. The white dot in each violin plot represents the median value of the proportion of different types of TIICs. (C) The different infiltration levels of six types of TIICs between different CNV types of *MSR1* and the *p*‐value of statistical difference were collected in Table S11. The dots with different colors represent the infiltration level of TIICs in different types of CNVs of *MSR1*. The middle line in the box plot represents the median value of infiltration level of TIICs, and the upper and lower lines in the box plot represent the upper quartile and lower quartile respectively. (D–F) Left: Cells in tumor tissue were categorized into 13 clusters. Middle: Blue dots represent the cells expressing *MSR1*. Right: Name of cells highly expressing *MSR1*. CNV, copy number variation; LGG, low‐grade glioma; TIICs, tumor‐infiltrating immune cells

The proportions of different types of TIICs in every sample are displayed in Figure 6A. The contents of several types of TIICs showed significant differences between the high and low expression groups of *MSR1*, such as Monocytes, Macrophages M1, and M2 (Figure 6B). Moreover, four types of TIICs (M0, M1, and M2 macrophages and activated memory CD4 T cells) had a positive correlation with *MSR1* (Figure S8A), while another four types of TIICs (eosinophils, activated natural killer [NK] cells, monocytes, and activated mast cells) had a negative correlation with *MSR1* (Figure S8B). The correlation within TIICs fluctuates greatly (Figure S8C). We also analyzed the prognostic value of six types of TIICs in predicting the cumulative survival rate for LGG patients (*p* < 0.001) (Figure S8D). In addition to macrophages, there are significant differences in the infiltration levels of the other five types of TIICs between different CNV types of MSR1 (Figure 6C, and Table S11). Lastly, single‐cell RNA‐seq data from glioma patients were processed and 13 types of cells were identified, including monocytes, macrophages, astrocytes, smooth muscle cells, and CD8+ T‐cells (Figure 6D,E). *MSR1* was mainly expressed in monocytes and macrophages (Figure 6F).

**FIGURE 7:**
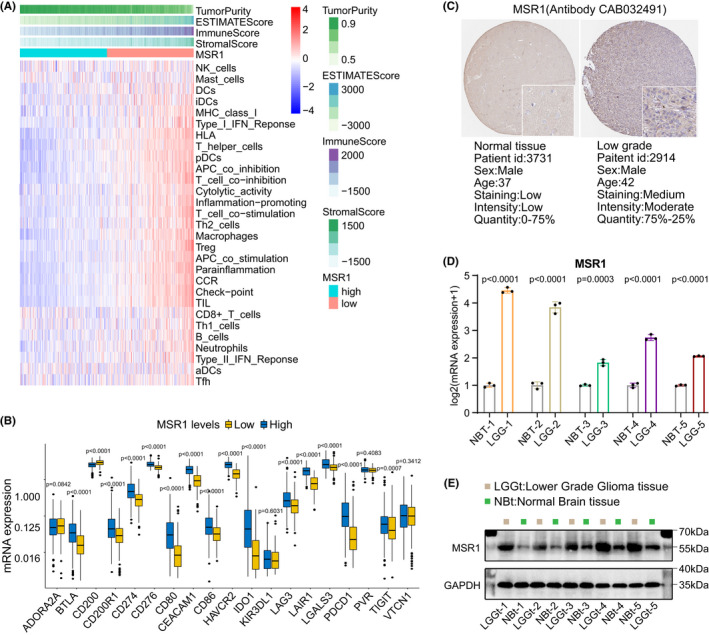
Association between *MSR1* and TME‐related score, TIICs, and ICPs. Experimental verification of the differential expression of *MSR1* between five pairs of LGG tissues and control normal brain tissues. (A) Difference analysis of enrichment score of 29 immune‐associated gene sets and four types of TME‐related score between high and low expression groups of *MSR1*. The *p*‐value of statistical difference was collected in Table S12. (B) Difference analysis of the expression level of ICPs between high and low expression groups of *MSR1*. Except for ADORA2A, KIR3DL1, PVR, and VTCN1, other ICPs had significant statistical differences between the two expression groups. The middle line in the box plot represents the median value of the expression level of ICP, and the upper and lower lines in the box plot represent the upper quartile and lower quartile respectively. (C) By comparing the intensity, density, and total quantity of immunohistochemical staining between the two types of tissues, it was found that the protein expression level of *MSR1* in LGG tissue was higher than that in control normal brain tissue. (D) Differential expression of *MSR1* at transcriptional level between LGG tissues and control normal brain tissues were measured by quantitative RT‐PCR. Except that the *p*‐value of statistical difference in the third pair of tissues is 0.0003, the *p*‐value in the other four pairs of tissues is less than 0.0001. The three black dots at the top of each histogram represent the CT value of the sample repeated three times. Data are presented as the mean ± SD. X‐axis: five pairs of samples. Y‐axis: expression level of *MSR1* (log2 transformed). (E) WB analysis for *MSR1* in LGG tissues and control normal brain tissues. In these five pairs of tissues, the protein expression level of *MSR1* in LGG tissue was significantly higher than that in control normal brain tissue. The leftmost and rightmost lanes correspond to markers, the lane corresponding to the gray box represents LGG tissue, and the lane corresponding to the green box represents normal brain tissue. The WB analysis was repeated three times independently. The results of the other two repetitions of WB can be seen in Figure S11B,C. ICP, immune checkpoint; IHC, immunohistochemistry; SD, standard deviation; TME, tumor microenvironment; WB, western blot

Furthermore, difference analysis of enrichment scores of immune‐associated gene sets and TME‐related scores between high and low expression groups of *MSR1* were performed in the TCGA cohort. There were significant differences in enrichment scores of most immune‐associated gene sets and four types of TME‐related scores between the two expression groups (Figure 7A), and the *p*‐value of statistical difference were collected in Table S12. All enrichment scores of immune‐associated gene sets had a positive correlation with *MSR1*, and the results were shown in the scatter plots (Figure S9). Finally, to assess the immunotherapeutic potential of *MSR1*, we explored the relationship between *MSR1* and ICPs and found that the expression levels of most ICPs were significantly different between the high and low expression groups of *MSR1* (Figure 7B), and most ICPs were positively correlated with *MSR1* (Figure S10).

### 3.7 Experimental part

Immunohistochemistry (IHC) data, retrieved from the HPA website, showed that the protein expression level of *MSR1* in LGG tissue was higher than that in control brain tissue (Figure 7C). The intensity, density, and total quantity of immunohistochemical staining in LGG tissue were higher than those in normal brain tissue. To further clarify the differential expression of *MSR1* between LGG tissue and control normal brain tissue at the transcriptional and translational levels, western blot (WB) analysis and qRT‐PCR were performed in five pairs of tissues, respectively. The results showed that the mRNA and protein expression levels of *MSR1* were higher in LGG tissue than that in brain tissue (Figure 7D,E). WB and qRT‐PCR were repeated three times independently.

## 4 DISCUSSION

Although there are several treatment strategies for glioma patients, including surgery, radiation, and chemotherapy, glioma still cannot be cured.^42^ Therefore, we need to find new therapeutic strategies through advanced research. The mechanism of tumor recurrence and progression is very complex. An accumulating body of research indicates that TME plays an important role in tumor immunotherapy. Transforming the TME from a tumor‐friendly environment to a tumor suppressor environment is an effective strategy for tumor treatment.^43^, ^44^ In recent years, the research on TME has become a hot spot in the field of tumor immunotherapy, especially the targeted therapy of ICPs has achieved good clinical therapeutic effects in some types of cancers.^45^, ^46^ Anti‐CTLA‐4 antibody alone or in combination with anti‐PD‐1 antibody significantly prolonged the long‐term survival of the immunocompetent murine glioblastoma model.^47^ However, compared with other types of tumors, LGG exhibits a relatively poor response to ICP inhibitor immunotherapy. At present, we are facing a series of great challenges, such as identifying specific drugs directed against new therapeutic targets, enhancing the immunotherapeutic response of LGG patients without causing immune‐related adverse reactions.

The *MSR1* gene was first found on chromosome 19. Subsequently, it was found on all chromosomes (but not in the mitochondrial genome).^48^ Most of the *MSR1* repeats are located close to the transcription start site, suggesting that the MSR1 repeats are associated with short‐term transcriptional regulation. The CNV of *MSR1* is likely to affect the occurrence and development of a variety of tumors. Recent studies have shown that *MSR1* is highly expressed in M2‐like pre‐tumor macrophages,^27^, ^49^, ^50^ which are associated with tumorigenesis and development, such as angiogenesis and immunosuppressive factor production.^51^, ^52^ In *MSR1*‐deficient mice, ovarian and pancreatic cancer development was significantly inhibited.^53^ A growing number of studies suggest that *MSR1* may play a role in macrophage‐induced tumor activation and act as a molecular switch to regulate gene expression. Our study aimed to identify the prognostic value and immune role of *MSR1* in LGG patients based on various databases and websites.

The prognostic value of *MSR1* in pan‐cancer was studied by using Cox regression analysis, the Kaplan–Meier method, and the log‐rank test. The results suggest that *MSR1* can be used as a prognostic biomarker for multiple types of tumors, including LGG. Three independent LGG cohorts were downloaded from TCGA and CGGA databases and analyzed comprehensively. In addition to gender, there were significant differences in the expression of *MSR1* between the other five clinicopathological characteristics in the three cohorts. Moreover, *MSR1* has a high prognostic value and can be used as an independent prognostic factor in LGG patients. At the same time, the expression level of *MSR1* in LGG tissue was significantly higher than that in normal brain tissue, which is confirmed by experiments. To get a better understanding of the role of *MSR1* in LGG, GO, and KEGG enrichment analysis of the DEGs revealed that *MSR1* was closely related to immune‐related functions and signal pathways. Additionally, GSEA identified several tumor‐related hallmark gene sets, such as the *P53* pathway, *P13K/AKT/mTOR* signaling pathway, apoptosis, and *TGF‐β* signaling pathway.

Based on the above research, we further analyzed the role of *MSR1* in the TME of LGG patients. TME‐related scores not only had a significant difference between high and low expression groups of *MSR1* but also had a significant correlation with *MSR1*. Similar results were obtained in the study of immune‐associated gene sets. It has been reported that if tumor cells are not completely removed by effector T cells in the early stage of tumorigenesis, chronic inflammatory reactions will occur that limit the function of T cells.^54^ We found similar results indicated that TAMs play an important role in initiating an anti‐inflammatory process, thus producing a TME conducive to tumor development to protect tumor cells from immune damage. By analyzing the correlation between *MSR1* and TIICs, we found that *MSR1* was positively correlated with the content of M1 and M2 macrophages and negatively correlated with the content of NK cells and monocytes. The above results suggest that *MSR1* may play an immunosuppressive role in the TME. As ICPs inhibition is an effective treatment for many types of cancer, we explored the relationship between *MSR1* and ICPs in LGG patients. The results showed that most ICPs had significant differences between the high and low expression groups of *MSR1*, and were positively correlated with *MSR1*, especially *PD‐1*, *PD‐L1*, *CD4*, *HAVCR2* (*TIM‐3*), *LAP3*, and *TGFB1*. *HAVCR2* can downregulate *STAT1* and promote the *TGF‐β* signaling pathway to trigger M2 macrophage polarization.^55^, ^56^ Further, macrophage and T‐cell functions can be rescued by blocking *HAVCR2*. These findings indicate that targeting *MSR1* and *HAVCR2* in LGG patients may be therapeutically effective, especially for patients who are resistant to inhibitors of *PD‐1/PD‐L1/CTLA‐4*.^57^

Although we have conducted extensive research on *MSR1*, there are still some limitations in this study. We do not have our sequencing data. If we have our independent validation queue, this study will be more convincing. In addition, we did not deeply study the specific biological mechanism of *MSR1* in TME, which will be the focus of our later research. In conclusion, this study comprehensively analyzed *MSR1* in LGG and found that *MSR1* is a potential prognostic biomarker. Moreover, *MSR1* may be involved in the changes of TME.

## CONFLICT OF INTEREST

The authors declare that the research was conducted in the absence of any commercial or financial relationships that could be construed as a potential conflict of interest.

## AUTHOR CONTRIBUTIONS

Xingen Zhu constructed this study; Qiankun Ji, Kai Huang, and Yuan Jiang performed the data analysis, figures plotted and writing; Kunjian Lei and Zewei Tu did the western blotting and polymerase chain reaction experiments. Haitao Luo was responsible for the data acquisition and critical reading of the manuscript. All authors have read and approved the final manuscript.

## ETHICAL APPROVAL STATEMENT

The study was allowed by the medical ethics committee. We obtained the consent of all the surgical patients.

## Supporting information


Figure S1

Figure S2

Figure S3

Figure S4

Figure S5

Figure S6

Figure S7

Figure S8

Figure S9

Figure S10

Figure S11

Table S1

Table S2

Table S3

Table S4

Table S5

Table S6

Table S7

Table S8

Table S9

Table S10

Table S11

Table S12

## Data Availability

The data analyzed in our study can be found in the TCGA (http://cancergenome.nih.gov/), CGGA (http://www.cgga.org.cn/), and UCSC Xena data portal (http://xena.ucsc.edu/).
