# Supplementary material for: Comprehensive analysis of the prognostic and role in immune cell infiltration of MSR1 expression in lower‐grade gliomas
Source: Cancer Med. 2022 Feb 10;11(9):2020–35. doi: 10.1002/cam4.4603 (PMC9089222; doi:10.1002/cam4.4603)
Supplement: Supplementary file 1 — Figure S1 [file CAM4-11-2020-s003.pdf]

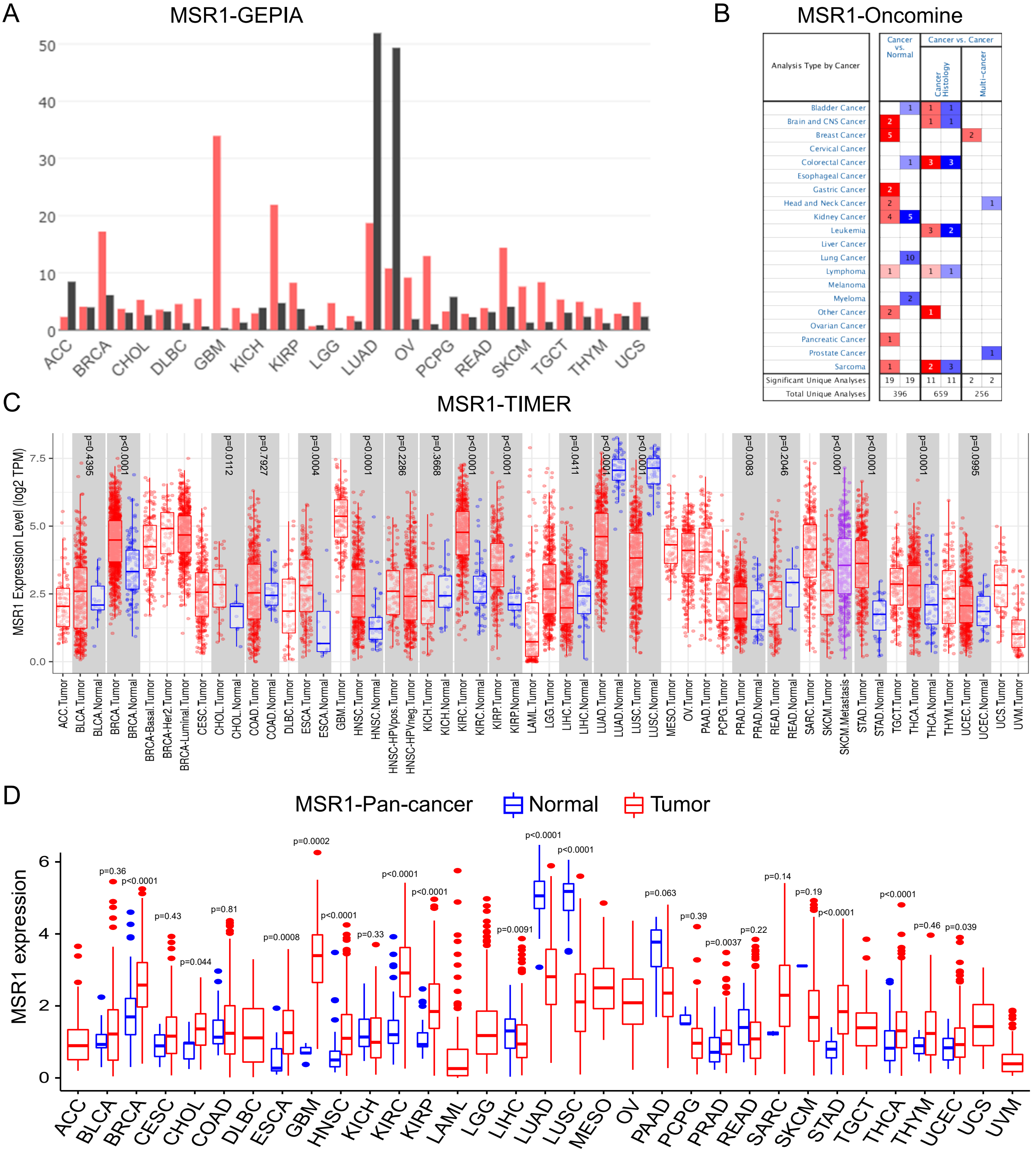

A
MSR1-GEPIA
0
10
20
30
40
50
ACC
BRCA
CHOL
DLBC
GBM
KICH
KIRP
LGG
LUAD
OV
PCPG
READ
SKCM
TGCT
THYM
UCS
B
MSR1-Oncomine
Analysis Type by Cancer
Cancer vs. Normal
Cancer vs. Cancer
Cancer Histology
Multi-cancer
Bladder Cancer
Brain and CNS Cancer
Breast Cancer
Cervical Cancer
Colorectal Cancer
Esophageal Cancer
Gastric Cancer
Head and Neck Cancer
Kidney Cancer
Leukemia
Liver Cancer
Lung Cancer
Lymphoma
Melanoma
Myeloma
Other Cancer
Ovarian Cancer
Pancreatic Cancer
Prostate Cancer
Sarcoma
Significant Unique Analyses
Total Unique Analyses
19 19 11 11 2 2
396 659 256
C
MSR1-TIMER
MSR1 Expression Level (log2 TPM)
0.0
2.5
5.0
7.5
p=0.4395
p<0.0001
p=0.0112
p=0.7927
p=0.0004
p<0.0001
p=0.2286
p=0.3668
p<0.0001
p<0.0001
p=0.0411
p<0.0001
p<0.0001
p=0.0083
p=0.2046
p<0.0001
p<0.0001
p<0.0001
p=0.0995
ACC.Tumor
BLCA.Tumor
BLCA.Normal
BRCA.Tumor
BRCA.Normal
BRCA-Basal.Tumor
BRCA-Her2.Tumor
BRCA-Luminal.Tumor
CESC.Tumor
CHOL.Tumor
CHOL.Normal
COAD.Tumor
COAD.Normal
DLBC.Tumor
ESCA.Tumor
ESCA.Normal
GBM.Tumor
HNSC.Tumor
HNSC.Normal
HNSC-HPVpos.Tumor
HNSC-HPVneg.Tumor
KICH.Tumor
KICH.Normal
KIRC.Tumor
KIRC.Normal
KIRP.Tumor
KIRP.Normal
LAML.Tumor
LGG.Tumor
LIHC.Tumor
LIHC.Normal
LUAD.Tumor
LUAD.Normal
LUSC.Tumor
LUSC.Normal
MESO.Tumor
OV.Tumor
PAAD.Tumor
PCPG.Tumor
PRAD.Tumor
PRAD.Normal
READ.Tumor
READ.Normal
SARC.Tumor
SKCM.Tumor
SKCM.Metastasis
STAD.Tumor
STAD.Normal
TGCT.Tumor
THCA.Tumor
THCA.Normal
THYM.Tumor
UCEC.Tumor
UCEC.Normal
UCS.Tumor
UVM.Tumor
D
MSR1-Pan-cancer
Normal
Tumor
MSR1 expression
0
2
4
6
p=0.36
p<0.0001
p=0.43
p=0.044
p=0.81
p=0.0008
p=0.0002
p<0.0001
p=0.33
p<0.0001
p<0.0001
p=0.0091
p<0.0001
p<0.0001
p=0.063
p=0.39
p=0.0037
p=0.22
p=0.14
p=0.19
p<0.0001
p<0.0001
p=0.46
p=0.039
ACC
BLCA
BRCA
CESC
CHOL
COAD
DLBC
ESCA
GBM
HNSC
KICH
KIRC
KIRP
LAML
LGG
LIHC
LUAD
LUSC
MESO
OV
PAAD
PCPG
PRAD
READ
SARC
SKCM
STAD
TGCT
THCA
THYM
UCEC
UCS
UVM
