# Supplementary figures and images for: Comprehensive analysis of the prognostic and role in immune cell infiltration of MSR1 expression in lower‐grade gliomas
Source: Cancer Med. 2022 Feb 10;11(9):2020–35. doi: 10.1002/cam4.4603 (PMC9089222; doi:10.1002/cam4.4603)

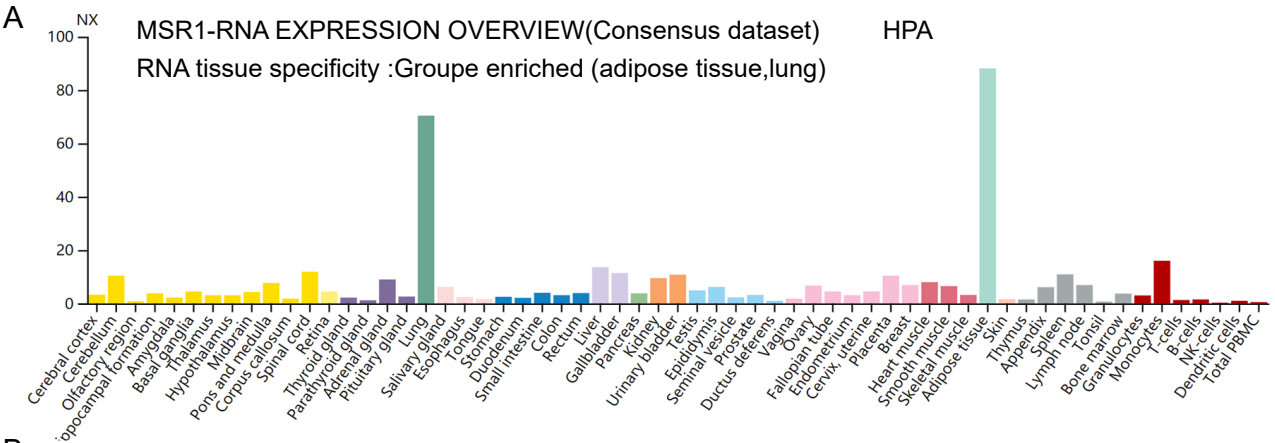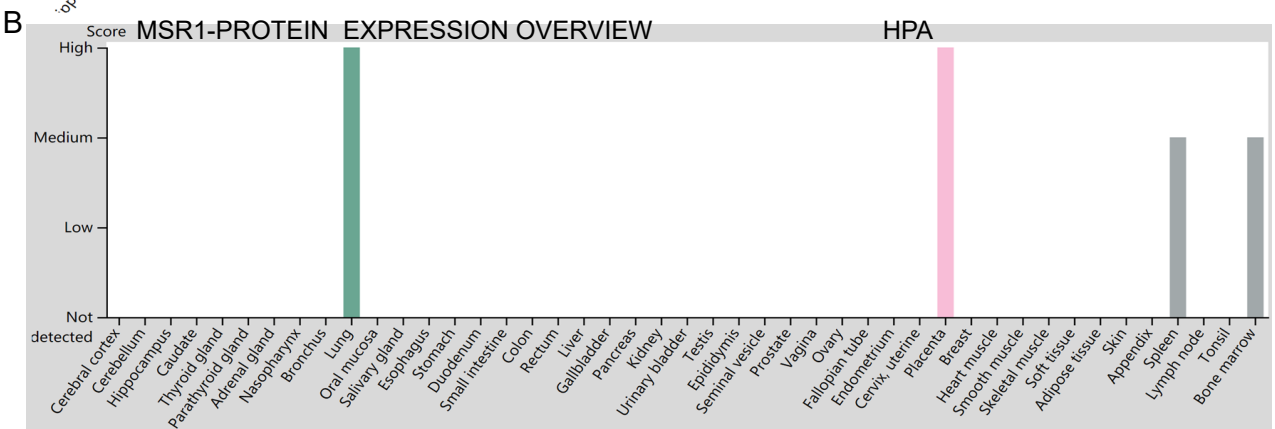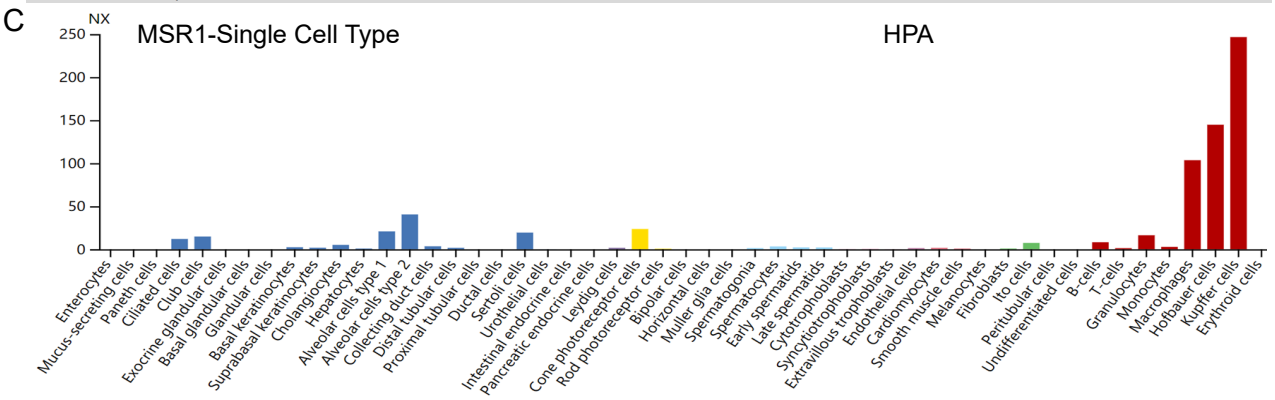

Supplement: Supplementary file 2 — Figure S2 [file CAM4-11-2020-s004.pdf]

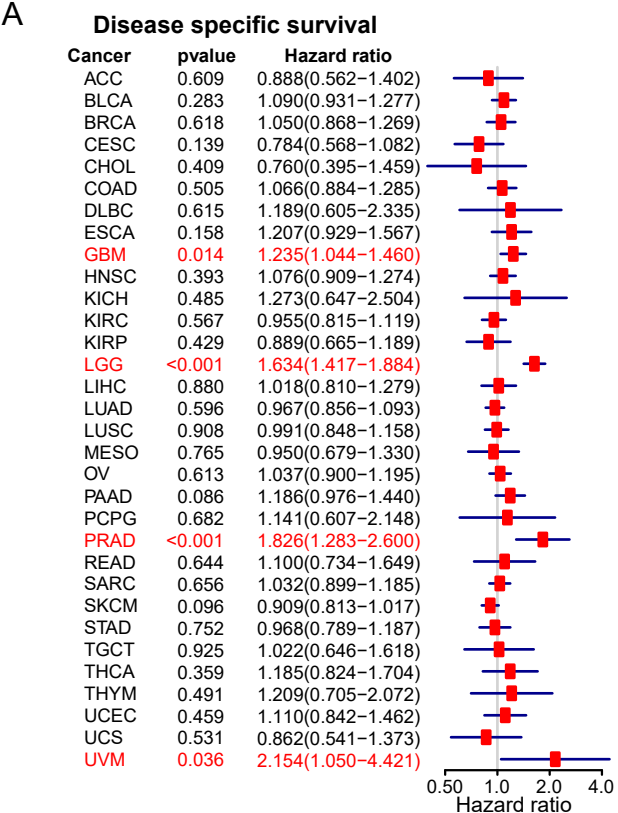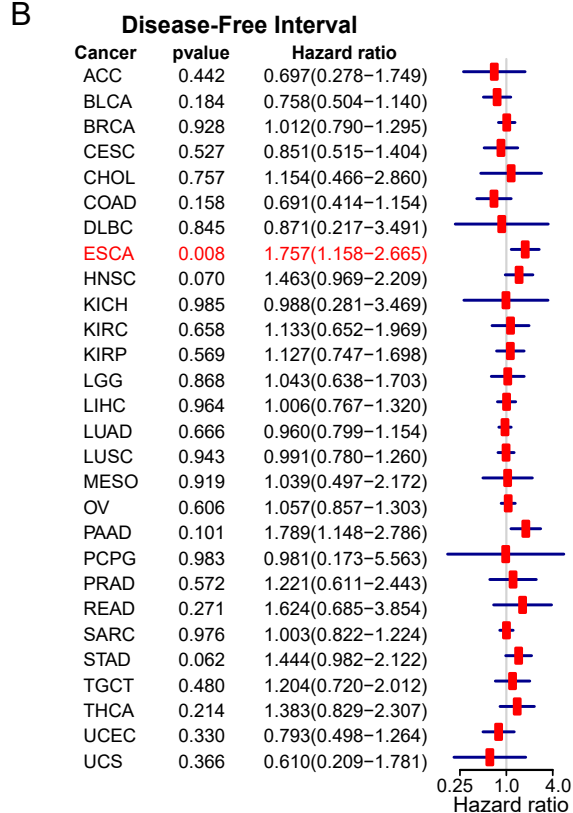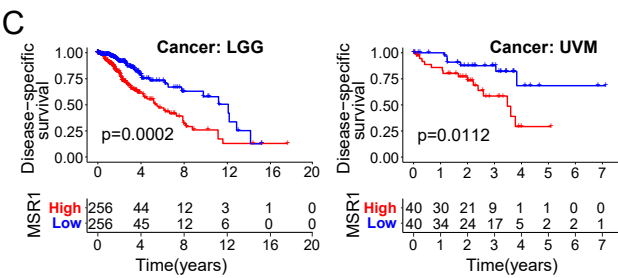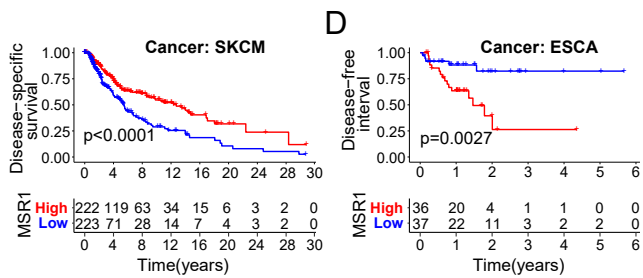

Supplement: Supplementary file 3 — Figure S3 [file CAM4-11-2020-s013.pdf]

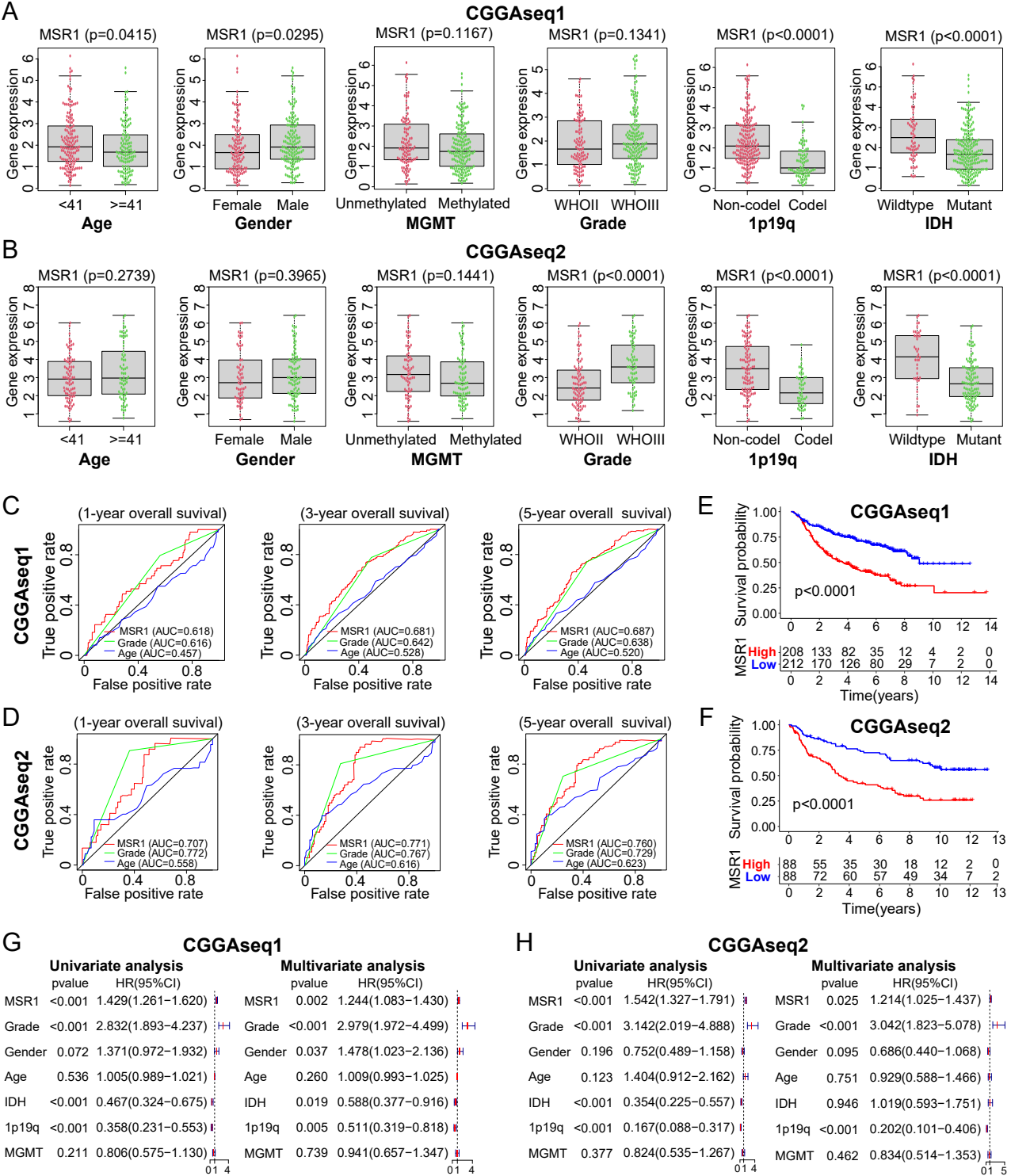

Supplement: Supplementary file 4 — Figure S4 [file CAM4-11-2020-s006.pdf]

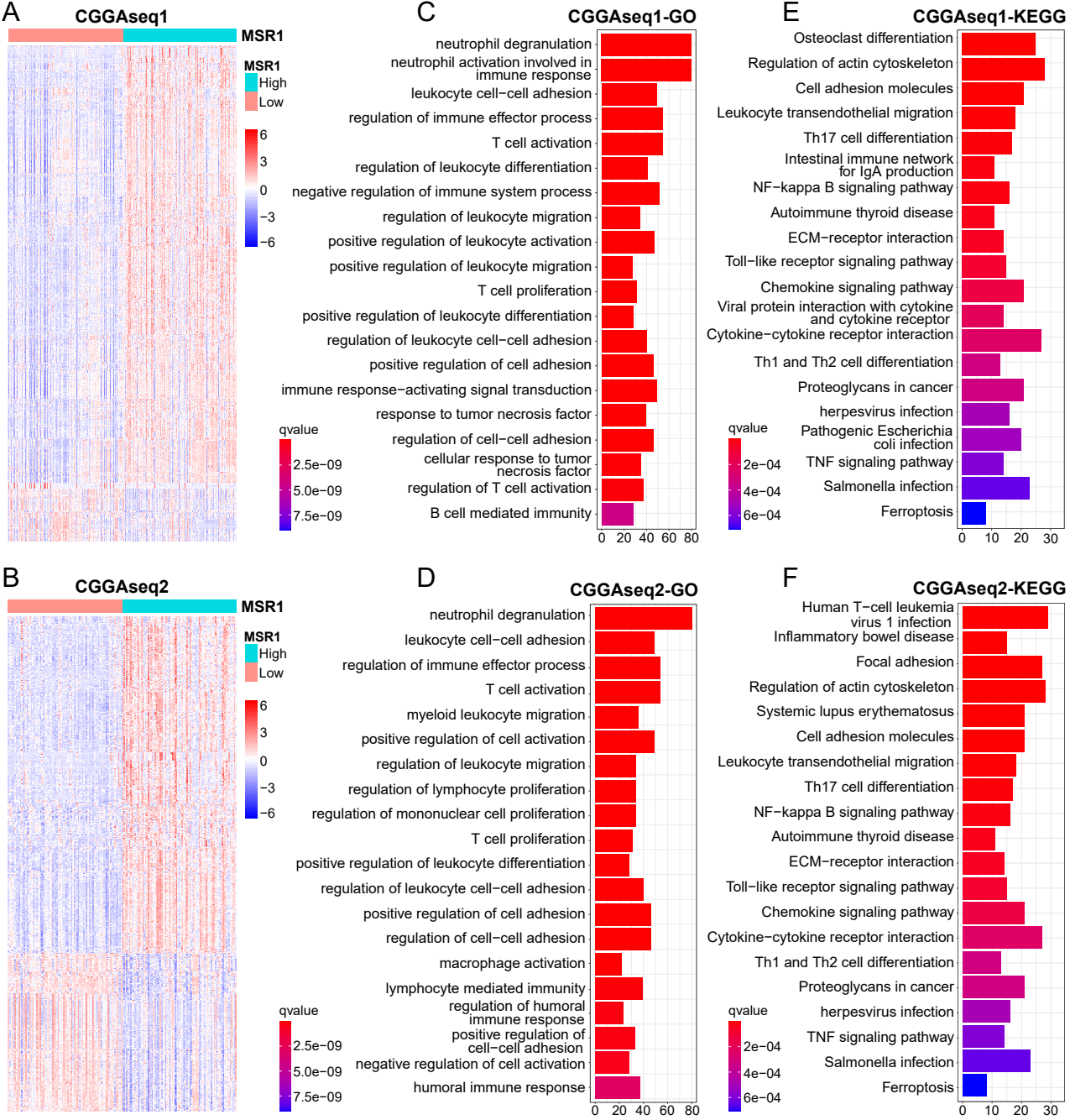

Supplement: Supplementary file 6 — Figure S6 [file CAM4-11-2020-s018.pdf]

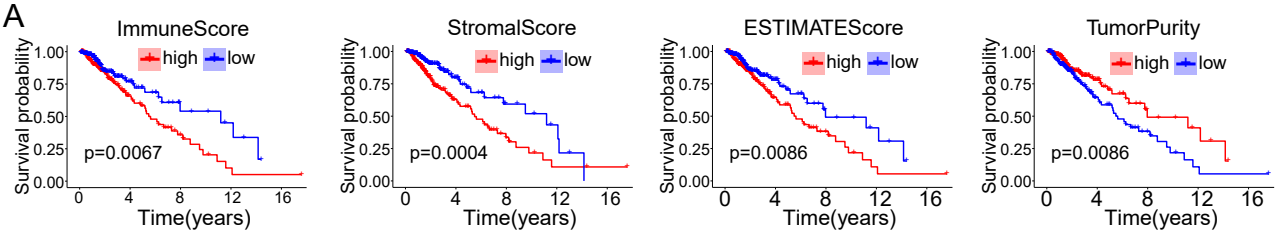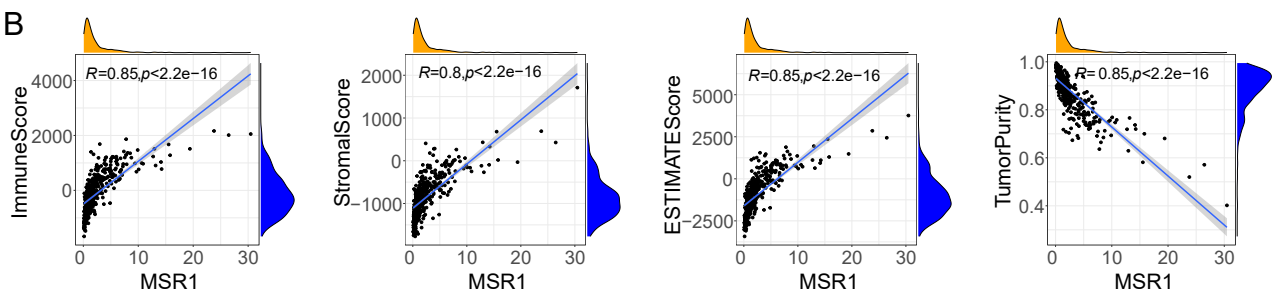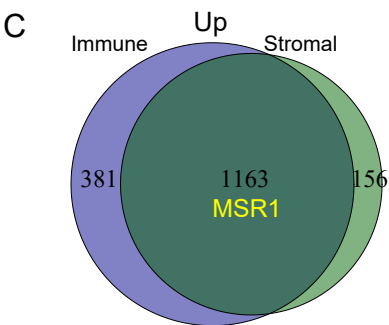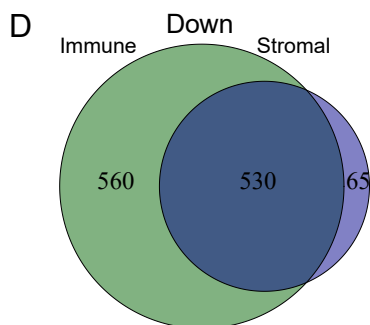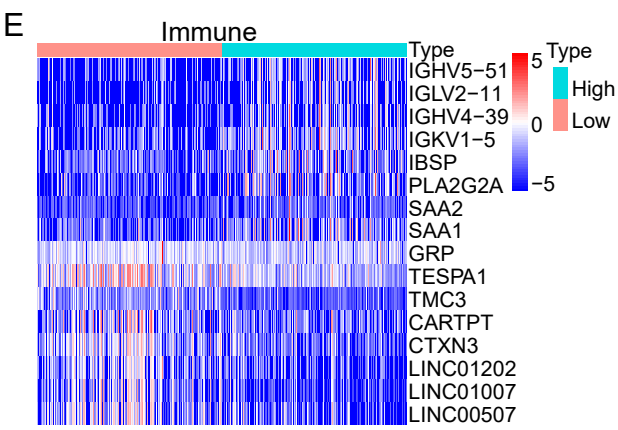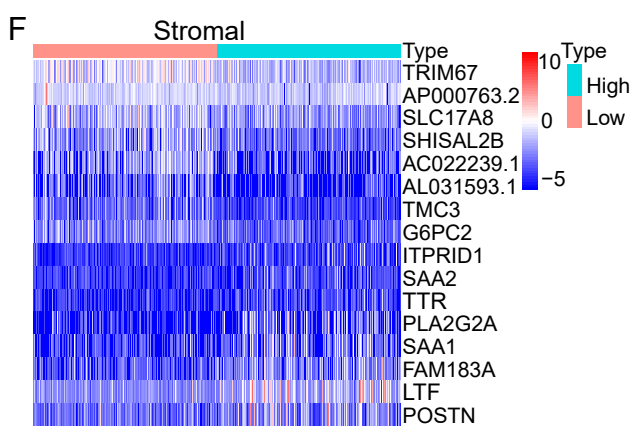

Supplement: Supplementary file 7 — Figure S7 [file CAM4-11-2020-s021.pdf]

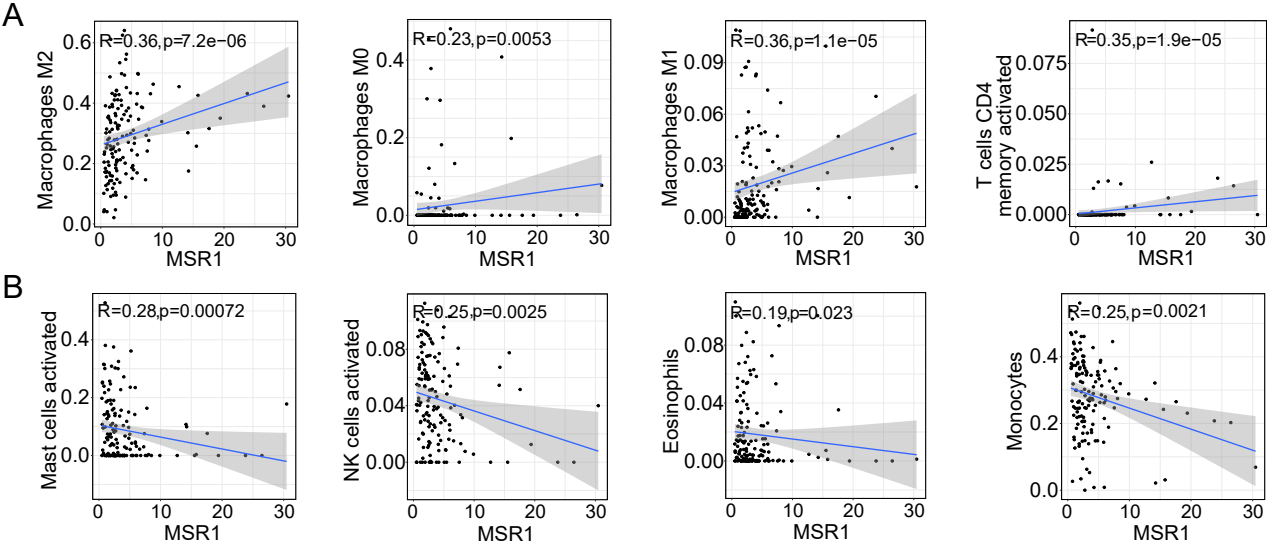

**C**

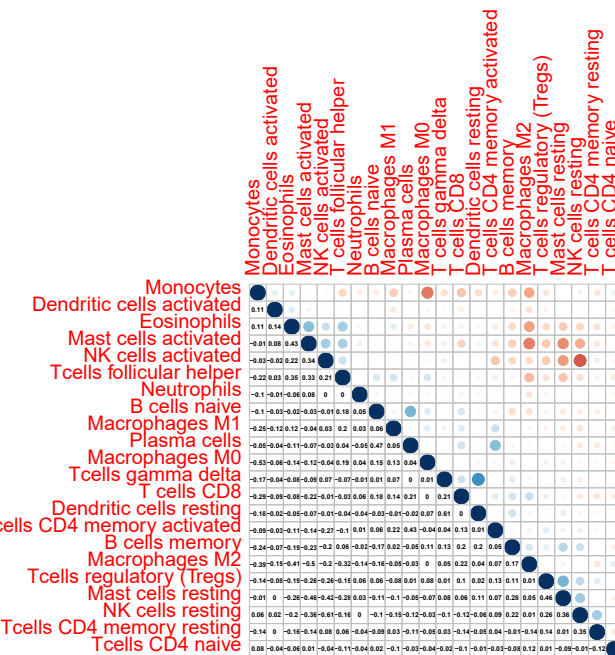

**D**

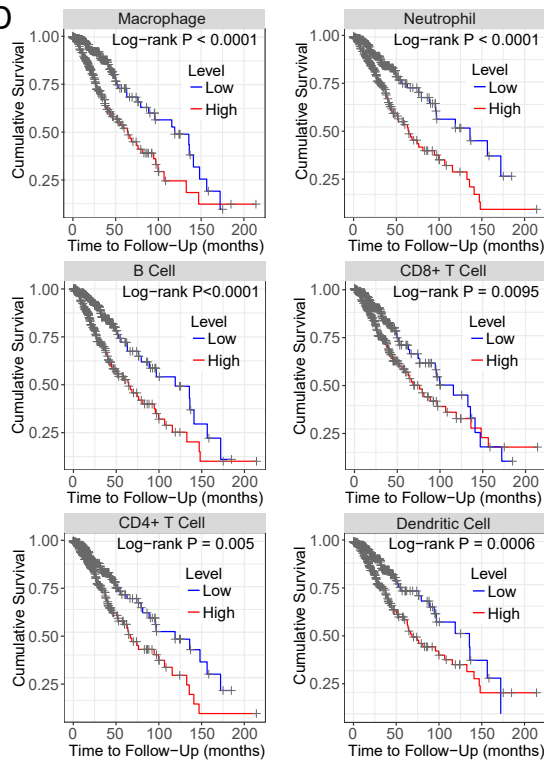

Supplement: Supplementary file 8 — Figure S8 [file CAM4-11-2020-s009.pdf]

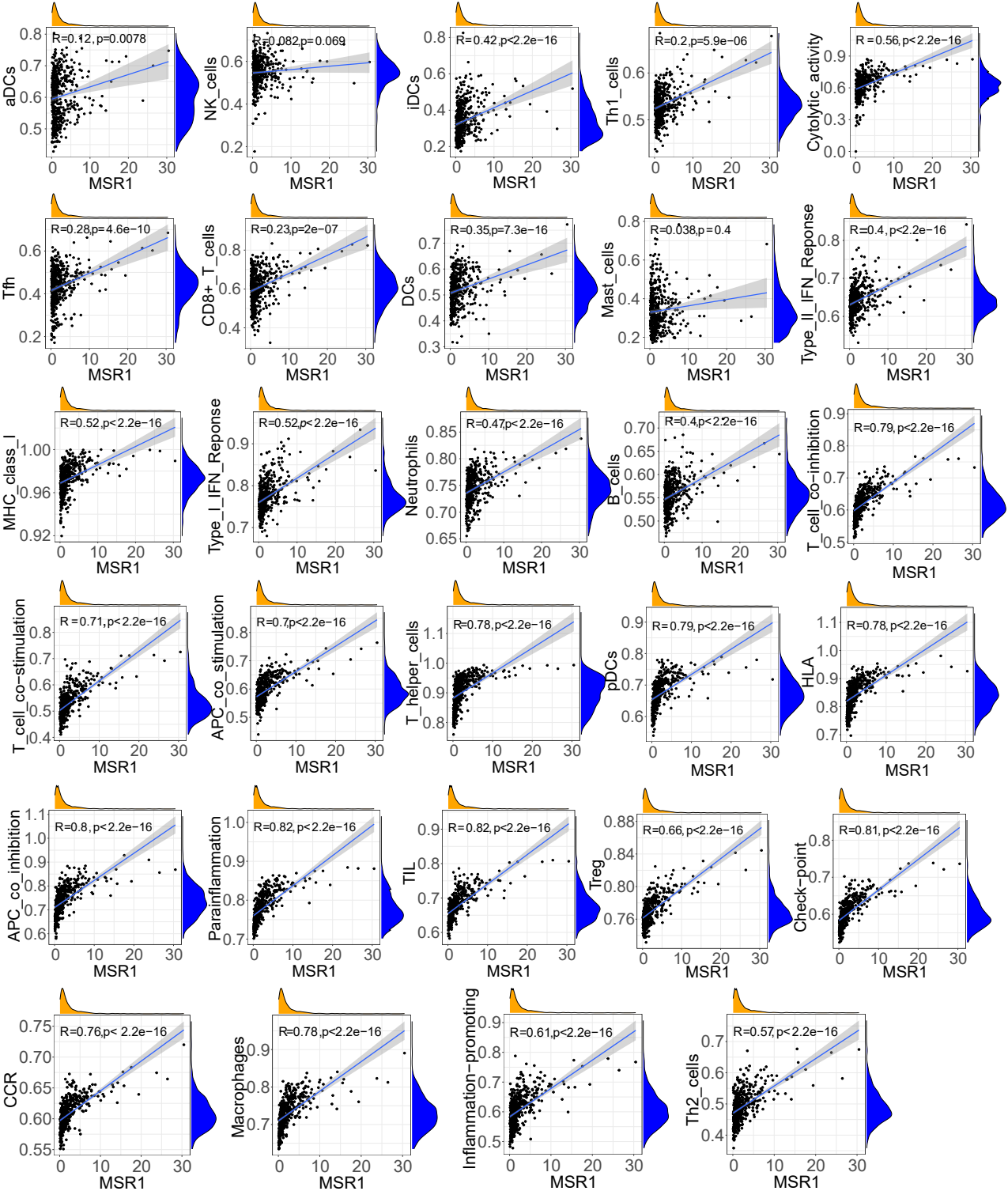

Supplement: Supplementary file 9 — Figure S9 [file CAM4-11-2020-s002.pdf]

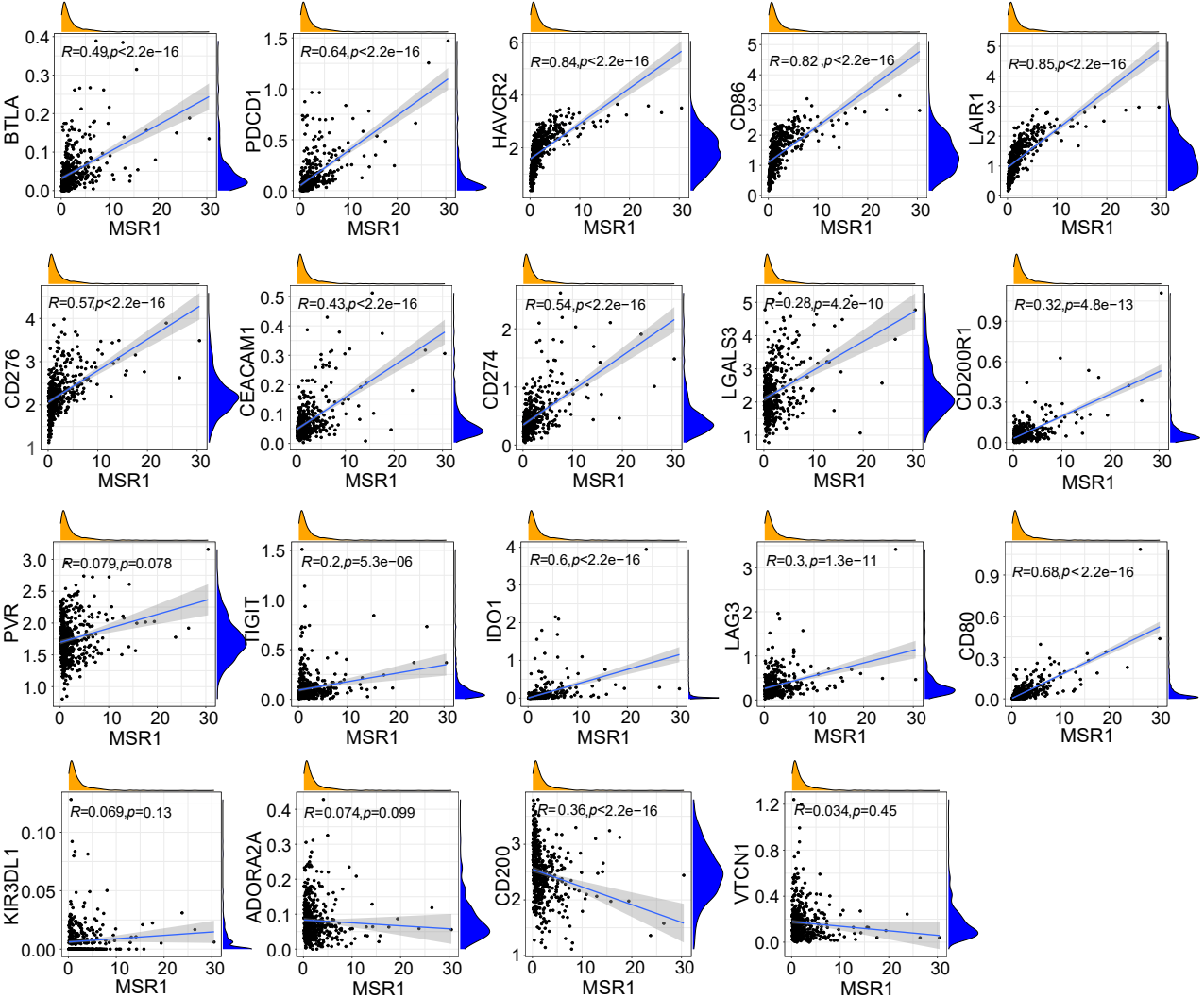

Supplement: Supplementary file 10 — Figure S10 [file CAM4-11-2020-s001.pdf]

A

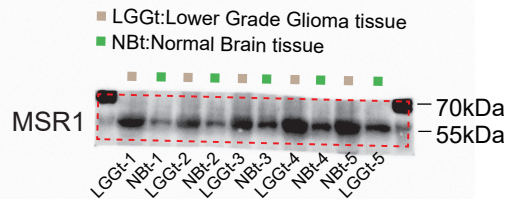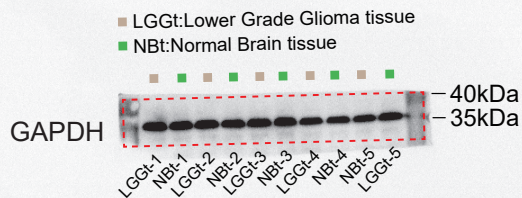

B

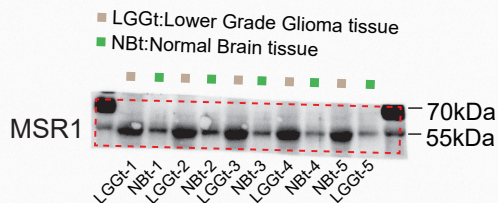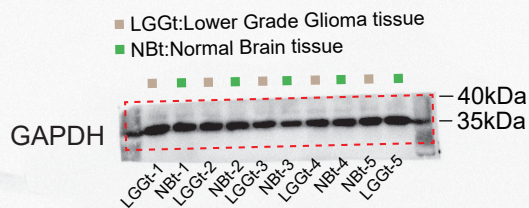

C

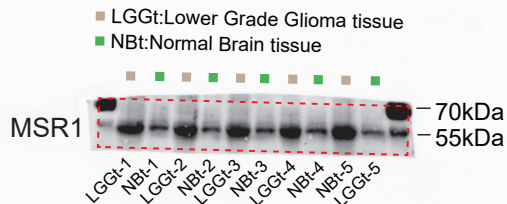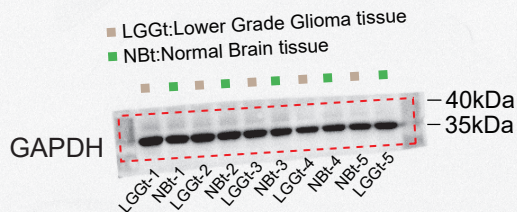

Supplement: Supplementary file 11 — Figure S11 [file CAM4-11-2020-s011.pdf]
