# Supplementary material for: Comprehensive analysis of the prognostic and role in immune cell infiltration of MSR1 expression in lower‐grade gliomas
Source: Cancer Med. 2022 Feb 10;11(9):2020–35. doi: 10.1002/cam4.4603 (PMC9089222; doi:10.1002/cam4.4603)
Supplement: Supplementary file 5 — Figure S5 [file CAM4-11-2020-s019.pdf]

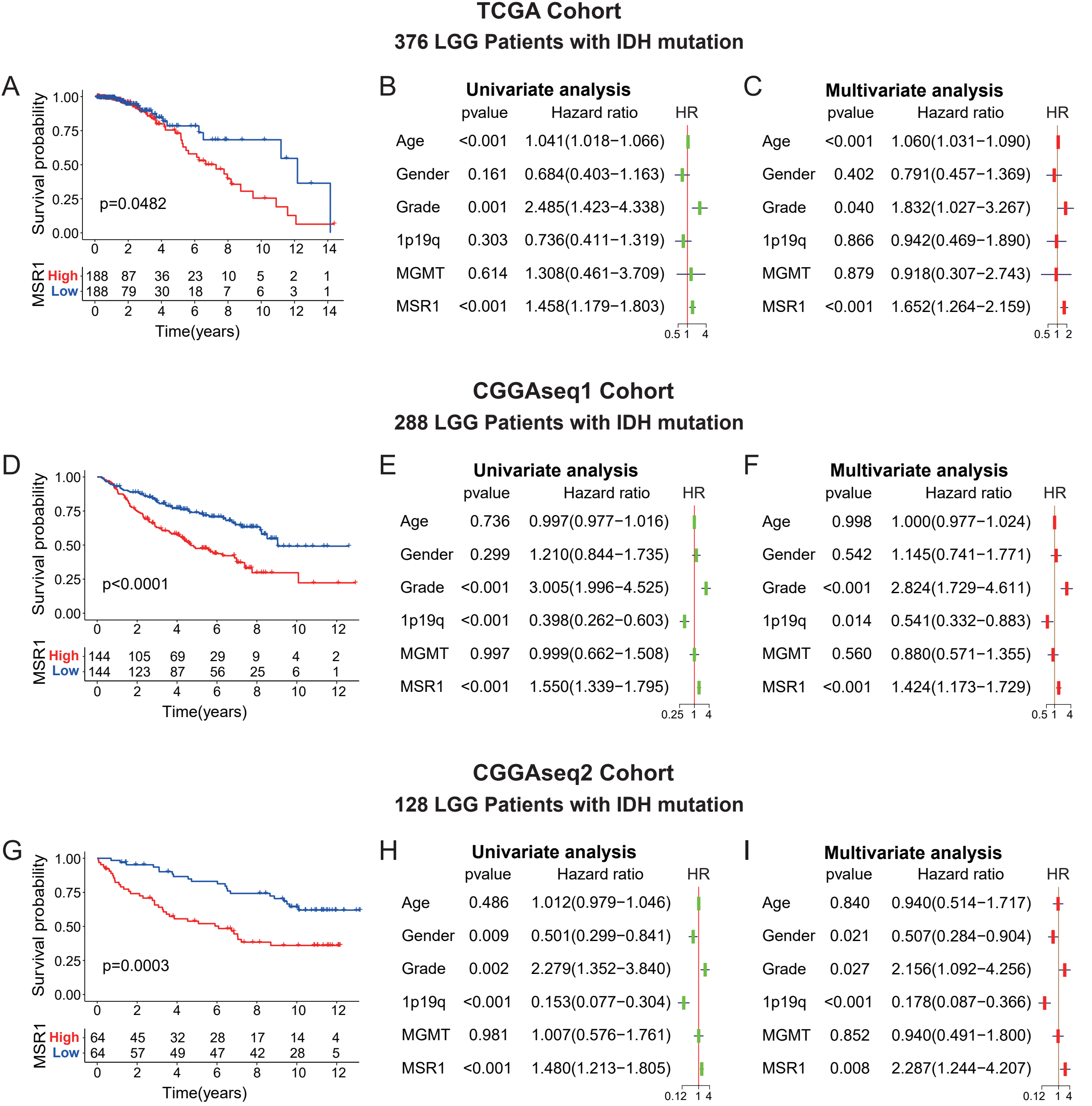

TCGA Cohort
376 LGG Patients with IDH mutation
A
Survival probability
1.00
0.75
0.50
0.25
0.00
p=0.0482
0 2 4 6 8 10 12 14
MSR1
High 188 87 36 23 10 5 2 1
Low 188 79 30 18 7 6 3 1
0 2 4 6 8 10 12 14
Time(years)
B
Univariate analysis
pvalue Hazard ratio HR
Age <0.001 1.041(1.018−1.066)
Gender 0.161 0.684(0.403−1.163)
Grade 0.001 2.485(1.423−4.338)
1p19q 0.303 0.736(0.411−1.319)
MGMT 0.614 1.308(0.461−3.709)
MSR1 <0.001 1.458(1.179−1.803)
0.5 1 4
C
Multivariate analysis
pvalue Hazard ratio HR
Age <0.001 1.060(1.031−1.090)
Gender 0.402 0.791(0.457−1.369)
Grade 0.040 1.832(1.027−3.267)
1p19q 0.866 0.942(0.469−1.890)
MGMT 0.879 0.918(0.307−2.743)
MSR1 <0.001 1.652(1.264−2.159)
0.5 1 2
CGGAseq1 Cohort
288 LGG Patients with IDH mutation
D
Survival probability
1.00
0.75
0.50
0.25
0.00
p<0.0001
0 2 4 6 8 10 12
MSR1
High 144 105 69 29 9 4 2
Low 144 123 87 56 25 6 1
0 2 4 6 8 10 12
Time(years)
E
Univariate analysis
pvalue Hazard ratio HR
Age 0.736 0.997(0.977−1.016)
Gender 0.299 1.210(0.844−1.735)
Grade <0.001 3.005(1.996−4.525)
1p19q <0.001 0.398(0.262−0.603)
MGMT 0.997 0.999(0.662−1.508)
MSR1 <0.001 1.550(1.339−1.795)
0.25 1 4
F
Multivariate analysis
pvalue Hazard ratio HR
Age 0.998 1.000(0.977−1.024)
Gender 0.542 1.145(0.741−1.771)
Grade <0.001 2.824(1.729−4.611)
1p19q 0.014 0.541(0.332−0.883)
MGMT 0.560 0.880(0.571−1.355)
MSR1 <0.001 1.424(1.173−1.729)
0.5 1 4
CGGAseq2 Cohort
128 LGG Patients with IDH mutation
G
Survival probability
1.00
0.75
0.50
0.25
0.00
p=0.0003
0 2 4 6 8 10 12
MSR1
High 64 45 32 28 17 14 4
Low 64 57 49 47 42 28 5
0 2 4 6 8 10 12
Time(years)
H
Univariate analysis
pvalue Hazard ratio HR
Age 0.486 1.012(0.979−1.046)
Gender 0.009 0.501(0.299−0.841)
Grade 0.002 2.279(1.352−3.840)
1p19q <0.001 0.153(0.077−0.304)
MGMT 0.981 1.007(0.576−1.761)
MSR1 <0.001 1.480(1.213−1.805)
0.12 1 4
I
Multivariate analysis
pvalue Hazard ratio HR
Age 0.840 0.940(0.514−1.717)
Gender 0.021 0.507(0.284−0.904)
Grade 0.027 2.156(1.092−4.256)
1p19q <0.001 0.178(0.087−0.366)
MGMT 0.852 0.940(0.491−1.800)
MSR1 0.008 2.287(1.244−4.207)
0.12 1 4
